# Development of functional hybrid scaffolds for wound healing applications

**DOI:** 10.1016/j.isci.2022.104019

**Published:** 2022-03-02

**Authors:** Rahimeh B. Attasgah, Brenda Velasco-Rodríguez, Alberto Pardo, Javier Fernández-Vega, Lilia Arellano-Galindo, Luis Carlos Rosales-Rivera, Gerardo Prieto, Silvia Barbosa, José Félix Armando Soltero, Morteza Mahmoudi, Pablo Taboada

**Affiliations:** 1 Grupo de Física de Coloides y Polímeros, Departamento de Física de Partículas, Facultad de Física e Instituto de Investigaciones Sanitarias, Universidad de Santiago de Compostela, Santiago de Compostela, Spain; 2 Departamento de Ingeniería Química, CUCEI, Universidad de Guadalajara, Guadalajara, Mexico; 3 Grupo de Biofísica e Interfases, Departamento de Física de Aplicada, Facultad de Física, Universidad de Santiago de Compostela, Santiago de Compostela, Spain; 4 Department of Radiology and Precision Health Program, Michigan State University, 766 Service Road, East Lansing, MI 48824, USA

**Keywords:** Health sciences, Medicine, Materials science, Biomaterials

## Abstract

Hybrid hydrogels composed of chitosan (CS) and hyaluronic acid (HA) and collagen (Coll) were prepared by polyelectrolyte complex self-assembly. These scaffolds displayed a good intermingling of the polymeric chains, with porosities above 80% and good interconnected structures with pore sizes lying between 30–115 μm. The ionic interactions between CS and HA make the scaffolds have larger storage modulus and longer LVR regions than their pure counterparts. Both quantities progressively decrease as the HA and Coll concentrations in the formulation rise. These hybrid hydrogels showed good swelling extents from ca. 420 to ca. 690% and suitable resistance to enzymatic degradation, which was slightly lower for scaffolds containing CS to larger extents or Coll in the formulation. All scaffolds were largely cytocompatible and allowed the proliferation of both mouse fibroblast and human keratinocytes with their infiltration inside, thus becoming optimal matrices for intended tissue engineering applications as well as transdermal drug delivery depots.

## Highlights

• Hybrid scaffolds were obtained by polyelectrolyte ionic self-assembly
• Scaffolds were largely porous with suitable pore sizes for cell proliferation
• Scaffolds showed exceptional swelling and good resistance to enzymatic attack
• They were nontoxic and enabled cell proliferation and infiltration inside the scaffold

## Graphical abstract

**Figure:**
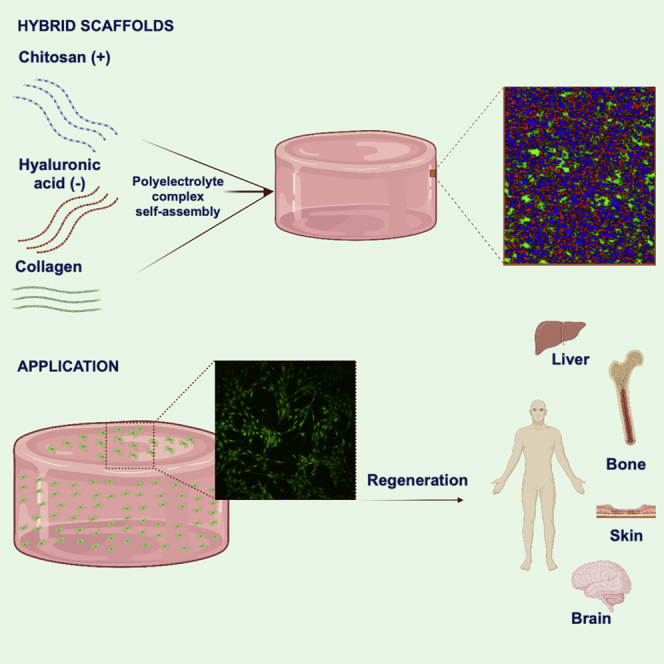

## Introduction

In response to the needs of tissue engineering (TE), new biomaterials with required features such as biocompatibility, bioresorption, bioactivity, and biodegradability to nontoxic byproducts have been designed (Elitok et al., 2018; Goriainov et al., 2014; Puppi et al., 2010). Biomaterials based on natural polymers are currently used to create porous scaffolds for filling tissue cavities and delivering bioactive compounds to salvage and/or regenerate tissues and restore their functionalities based on the characteristics of the tissue of origin (Lee et al., 2014; Ma et al., 2003; Mahmoudi et al., 2017; Naahidi et al., 2017) or to build up new artificial structures mimicking the *in vivo* microenvironment as model platforms to more reliable *in vitro* analyses and screening of therapeutics (Bissell and Hines, 2011; Hutmacher, 2010; Hutmacher et al., 2010; Kimlin et al., 2013).

The use of natural polymers, or biopolymers, in scaffolding as proteins (e.g., silk, fibroin, collagen, and gelatin) and polysaccharides (e.g., chitosan, carrageenan, chondroitin sulfate, sodium alginate, and hyaluronic acid) is based on their wide availability, economical price, biocompatibility, physiochemical properties similar to those of natural extracellular matrix (ECM), and biodegradability, among others (Burdick and Stevens, 2005; Chandra and Atala, 2019; Eke et al., 2017; Loessner et al., 2016; Saeedi Garakani et al., 2020a). Among polysaccharides, chitosan (CS) is a linear, seminatural polysaccharide derived from chitin through alkali deacetylation, which consists of N-glucosamine and N-acetylglucosamine units connected through (1–4)-glycosidic bonds (Hamedi et al., 2018). This is a very hydrophilic, inexpensive, and readily available polymer that shows outstanding biocompatibility and biodegradability as well as enhanced antimicrobial and antioxidant activities (Pellá et al., 2018; Saeedi Garakani et al., 2020b; Shen et al., 2021). Furthermore, the cationic nature of CS provides excellent mucoadhesive properties, an exceptional gel-forming ability (Hamedi et al., 2018), and can be served as a glycosaminoglycan (GAG) analogue in stimulating cellular processes, for example, in bone, cartilage, and skin regeneration processes (Hamedi et al., 2018; Pellá et al., 2018). However, for certain TE applications, chitosan-based scaffolds may lack sufficient mechanical strength, excessively long bioresorption times, and cannot provide a friendly interface for cells due to the absence of recognition and signaling motifs that facilitate cellular adhesion, proliferation, and/or differentiation processes for tissue regeneration (Kumar et al., 2017).

To create more appropriate scaffolds for directing and controlling the desired cell behaviors and to mechanically strengthen the engineered tissues, mixtures of CS with one or more biopolymers (e.g., hyaluronic acid [HA] and collagen [Coll]) are being used (Atashgah et al., 2021; Lu et al., 2013; Mellati et al., 2016; Saeedi Garakani et al., 2020b). HA is another natural anionic GAG polymer typically found in synovial fluid, skin, cartilage, and brain ECM, which holds a high capacity for water absorption and retention and plays a significant role in the assembly of extracellular and pericellular matrices by regulating porosity and malleability (Schanté et al., 2011) and acts as an environmental cue to regulate cell behavior during embryonic development, healing processes, and inflammation (Knudson, 2003). It also influences several cellular functions such as cell adhesion, migration, and proliferation related to homeostasis and tissue remodeling processes (Muzzarelli et al., 2012; Tognana et al., 2007). The combination of cationic chitosan with negatively charged HA allows the formation of polyelectrolyte complexes (PECs) through ionic bonding (Berger et al., 2004), which enhances scaffold stability, improves cell adhesion, and increases the mechanical strength of the resulting material. CS-HA hydrogels obtained in this way have been used in the form of 2D and 3D porous matrices for dental pulp (Coimbra et al., 2011; Miranda et al., 2016); abdominal (Deng et al., 2017), cartilage (Correia et al., 2011; Huang et al., 2019; Karabiyik Acar et al., 2018), and bone (Li et al., 2020) tissue regeneration; wound healing (Sanad and Abdel-Bar, 2017), and to configure 3D *in vitro* matrices to evaluate dose-dependent drug responses and gene expressions in tumor-mimicking environments as, for example, in the case of glioblastoma (Erickson et al., 2018; Wang et al., 2016).

On the other hand, Coll is another biopolymer widely used for scaffolding, as it is the main component of the connective tissue, responsible for the strength of skin, bones, tendons, and cartilage tissues (Lin et al., 2011). Their incorporation in scaffolds provides specific cell adhesion sites such as arginine–glycine–aspartate (RGD) sequences, which help to promote cellular adhesion, growth, and differentiation (Liu et al., 2019). Nevertheless, its rapid biodegradation and low mechanical strength make necessary its use in combination with other biopolymers such as CS and/or HA. In this regard, Coll-CS hybrid scaffolds have been tested as potential biomaterials for wound healing and skin regeneration (Udhayakumar et al., 2017; Yang et al., 2021; You et al., 2017), treatment of traumatic brain injury (Yan et al., 2019), neural axon recovery (Sun et al., 2019), vascularization modeling (Yin et al., 2020), myocardial TE (Fang et al., 2020), and bone repair (Kozlowska et al., 2019; Păun et al., 2020). Meanwhile, CS-HA-Coll scaffolds have proved to favor osteoinduction in bone restoration (Li et al., 2021) and to control stem cell differentiation and guidance for spinal cord repair (Liu et al., 2021).

Despite the extensive research analyzing the potential of pure and hybrid CS-based scaffolds in TE applications, poor attention has been made for understanding the correlation of scaffolds composition and concentration with the resulting biophysical and biochemical properties for intended TE applications. Hence, in this work, we aim to design new materials to obtain 3D polymeric matrices of pure CS or combined with HA and Coll and analyze the role of biopolymer molecular weight and concentration and matrix composition on the structure and physico-chemical properties of the resulting scaffolds. These were obtained by means of a simple polyionic-based self-assembly process. The obtained materials were characterized by a set of techniques in terms of composition, physicochemical, morphological, and mechanical properties as well as *in vitro* behavior, observing differences that are expected to influence their biological performance. Biological assays regarding viability, cell adhesion, and proliferation were assessed on model mouse Balb fibroblast and human dermal keratinocytes cells. The excellent biocompatibility of the polymeric matrices was confirmed by allowing important cell proliferation and cell spreading along the hydrogel matrices. The present findings suggested that the structure and properties can play a key role in the final application of the present scaffolds as effective matrices for TE, tentatively to guide, for example, dermal (e.g., wounds and dermal fillers), cartilage, and/or bone regeneration applications.

## Results and discussion

### Structural and thermodynamic characterization of scaffolds

#### Effect of CS molecular weight and concentration

Hybrid CS-HA and CS-HA-Coll hydrogel scaffolds of suitable composition were prepared by PEC after mixing of the biopolymeric solutions of CS and HA of different molecular weights followed by Coll incorporation as described when corresponding. When blended, CS and HA (and Coll) form a stable, PEC scaffold, which should combine the positive attributes of the respective materials and recapitulate and/or mimic, at least partially, the structural and physical properties of, for example, brain ECM (Erickson et al., 2018), skin (Feng et al., 2021) and cartilage tissues (Erickson et al., 2019; Mohan et al., 2017).

For the initially developed hybrid 3% XCS-1% YHA scaffolds, gel fraction lies in the range of 70%–80% (see Table 2). This quantity and the hydrogel density importantly decrease as the molecular weight of the CS used to form the hybrid scaffolds decreases, whereas the opposite trend is observed when the CS concentration in the composition raises.

**Figure 1:**
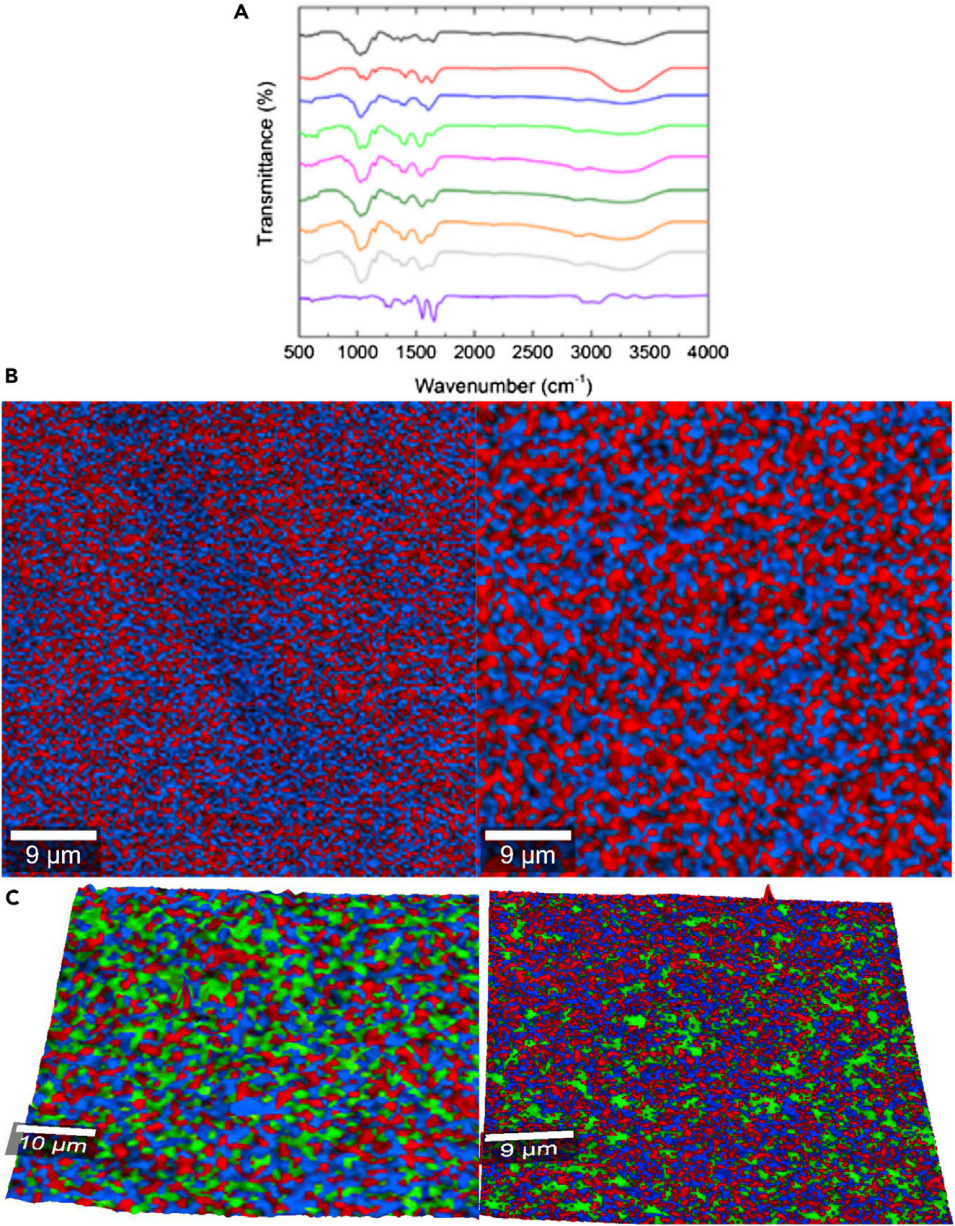
Physico-chemical and structural properties (A–C) (A) FTIR spectra of selected pure 3% HCS (), 3% LCS (), 1% HHA (), 3% HCS-1% HHA (), 4% HCS-1% HHA (), 3% HCS-2% HHA (), 3% HCS-2% HHA-1% Coll (), 3% HCS-2% HHA-2% Coll () hybrid hydrogels, and pure Coll (). Surface topographical composition acquired by Raman confocal imaging of (B) 2D projections of 3% HCS-1% HHA (left) and 3% HCS-2% HHA (right) hybrid hydrogels; (C) 3D projections of 3% HCS-1% HHA-1% Coll (left) and 3% HCS-1% HHA-2% Coll (right) hybrid ones. HHA (in red) was detected using bands at ca. 795 and 945 cm^−1^, HCS (in blue) using that of ca. 1540–1590 cm^−1^, and Coll that of ca. 1648 cm^−1^, respectively.

The scaffolds' structures were initially analyzed by FTIR after interpenetration and complexation of the individual polymer chains. The FITR spectrum of hybrid 3% HCS-1% HHA scaffold resembled the characteristic GAG structure of both CS and HA biopolymers as observed in Figure 1A, where the spectra of pure 3% HCS and 1% HHA are also shown. Broadband at ca. 3454–3025 cm^−1^ is attributed to O-H and N-H stretching vibrations of the functional groups engaged in intramolecular hydrogen bonding between CS and HA molecules (Pradal et al., 2011), whereas the complex band at 2972–2845 cm^−1^ corresponds to symmetric and asymmetric C-H stretching (Coimbra et al., 2011). In the fingerprint region of the spectrum, amide and carbonyl vibrations result in several distinctive bands between 1500 and 1700 cm^−1^: the hybrid 3% HCS-1% HHA scaffold shows the C=O stretching band located at ca. 1640 cm^−1^, hence, shifted compared with that of pure CS one (ca. 1649 cm^−1^), as the asymmetric stretching vibration of the carbonyl group COO^−^ of the carboxylate in HA is located at ca. 1606 cm^−1^ and overlaps an amide I shoulder (Coimbra et al., 2011). The overlapping band corresponding to amide II and N-H bending vibration of the deacetylated amine groups of CS in the hybrid scaffold intensifies and shifts to lower wavelengths (ca. 1539 cm^−1^ instead of 1566 cm^−1^ and 1559 cm^−1^ in pure CS and HA, respectively) due to the presence of HA in the composition, thus confirming the existence ionic interactions between CS and HA polymeric chains (see Figure 1A for details) (Florczyk et al., 2013). Bands at 1405–1381, 1308, 1152, 1067–1019, and 895 cm^−1^ from CH_2_ and CH_3_ deformation vibrations, N-H and C-N vibrations of amide III, asymmetric stretching of the C-*O*-C bridge, C-O stretching vibration, and β-glucosidic linkages of CS between glucose units, respectively (Olad and Azhar, 2014; Smitha et al., 2005), were also noted. In particular, the peak at 1405–1381 cm^−1^ becomes more prominent in the hybrid 3% HCS-1% HHA matrix (and also in 4% HCS-1% HHA) than in the corresponding pure CS ones indicative of CS-HA interactions, as previously reported (Erickson et al., 2018).

Neither CS concentration nor CS molecular weight involved significant differences in the FTIR spectra of hybrid CS-HA scaffolds (Figure 1A). Larger CS concentrations in the scaffold composition (i.e., 4% HCS-1% HHA) only slightly enhances the intensity of characteristic bands corresponding to C=O stretching and N-H bending vibrations at ca. 1640 and 1548 cm^−1^ as well as the one corresponding to asymmetric stretching of the C-*O*-C bridge at 1153 cm^−1^. The influence of HA in the spectrum of 4% HCS-1% HHA hydrogel is more screened than for 3% HCS-1% HHA, one consequence of the larger difference in components' concentration, as also observed in density and Young moduli (see Table 2 below). On the other hand, the use of CS of much lower molecular weights seems to favor the interactions between CS and HA chains as observed from a shift of the C-O stretching band to ca.1638 cm^−1^, which is converted to a shoulder, and the increase and shift of the band corresponding to N-H and C-N vibrations of amide III to ca. 1323 cm^−1^. Hence, these changes in the spectrum again suggest the formation of a chitosan-HA PEC due to the ionic interaction between the negatively charged carboxyl group (-COOH) of HA and the positively charged amino group (-NH_2_) of CS.

High-resolution confocal Raman images also denoted the suitable mixing of the polymers, without the existence of chain segregation. Figure 1B demonstrates the combined maps of the individual HA and CS polymers in the hybrid hydrogels, which were identified using some specific Raman bands of the biopolymers: 795 and 945 cm^−1^ for HA and ca. 1540–1590 cm^−1^ (amide I) for CS, respectively. As observed, the stability and preservation of the two initial components, HA and CS, are largely achieved. Both polymers are well distributed along with a coherent and homogeneous matrix, with HA chains well dispersed in the CS matrix. The good mixture of both types of polymers in the hybrid gel matrix can be considered a consequence of a successful complexation and intermingling of biopolymeric chains, proof of the existence of electrostatic interactions between the polymers. Interestingly, no interference was observed and, as a result, the surface topography of the scaffold was clearly resolved when performing a 3D projection of the images, as displayed here.

**Figure 2:**
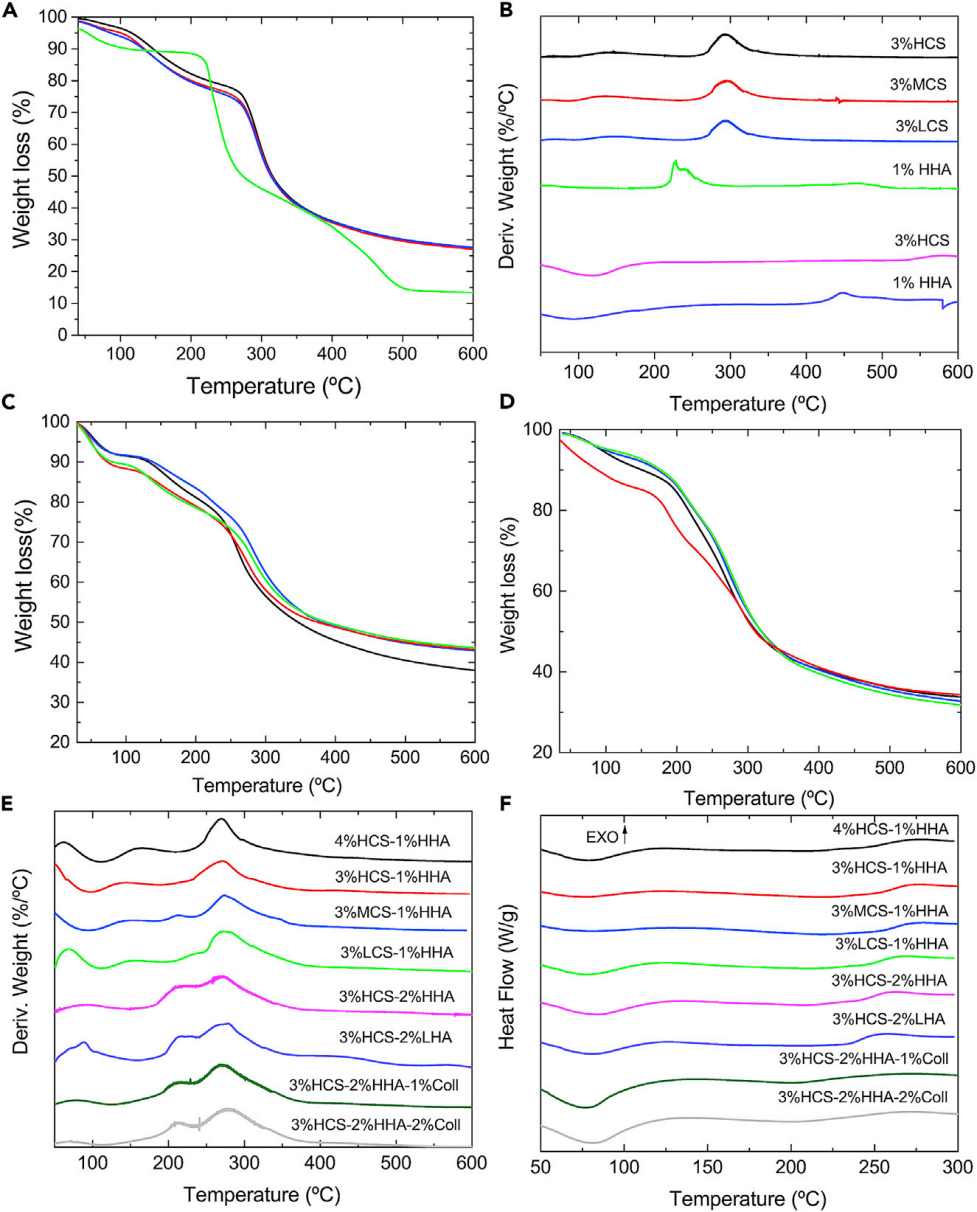
Thermal characterization (A–E) (A) Weight loss and (B) first derivative of weight loss for 3% HCS (), 3% MCS (), 3% LCS (), 1% HHA () pure hydrogels; (C) TGA plots for 3% HCS-1% HHA (), 4% HCS-1% HHA (), 3% MCS-1% HHA (), 3% HCS-1% LHA () hybrid hydrogels; (D) TGA plots for hybrid hydrogels with compositions 3% HCS-2% HHA (), 3% HCS-2% LHA (), 3% HCS-2% HA-1% Coll (), and 3% HCS-2% HHA-2% Coll (); (E) first derivatives of TGA data for the different hybrid scaffolds; (E) examples of DSC curves corresponding to different hybrid hydrogel scaffolds.

TGA data of hybrid 3% HCS-1% HHA, 3% HCS-1% MHA, 3% HCS-1% LHA, and 4% HCS-1% HHA scaffolds (Figure 2C) show that weight losses take place in three main steps as occurred for pure HA and CS ones (Figures 2A and 2B). The first step is assigned to the loss of residual water molecules in the samples within the temperature range 30–140°C, with a 10%–15% weight loss depending on the CS concentration and molecular weight. Polysaccharides and proteins have an important affinity for water molecules with different interaction strengths: the free water released and water linked through hydrogen bonds at ca. 40–120°C (Ding et al., 2013; Kumar et al., 2012) and water strongly bound through polar interactions to carboxylate groups released at ca. 140–160°C (Pieróg et al., 2009). The next decomposition steps took place between ca. 200–350°C and 350–600°C with total weight losses in the range of 47%–51% and 56%–62%, respectively, which correspond to the thermal and oxidative decomposition of CS and HA. Decomposition extents of pure and hybrid CS-HA hydrogel are shown in Table 1 (see also Figures 2C and 2D).

**Table 1:** Extents of scaffold degradation at the different stages are denoted from TGA measurements

Samples	Decomposition (%)	
	350°C	600°C
3% HCS	∼60	∼73
1% HHA	∼61	∼86
3% HCS-1% HHA	∼46	∼57
3% MCS-1% HHA	∼48	∼56
3% LCS-1% HHA	∼49	∼57
4% HCS-1% HHA	∼51	∼62

From Table 1, it can be observed that hybrid matrices showed smaller decompositions than pure ones. Also, when medium- or low-molecular-weight CS is used in the formulation, the lower biopolymer viscosity should favor a better diffusion of CS chains upon hydrogel formation, thus favoring electrostatic interactions with HA chains, which should help in stabilizing the structure, as confirmed from a very slight decrease in thermal decomposition in the temperature range 40–375°C but within the experimental uncertainty; other attractive interactions such as hydrogen bonding and hydrophobic interactions might also play a stabilization role too. This behavior is different from that observed for pure CS scaffolds, for which the use of MCS and HCS involved lower thermal stabilities in the range 30–350°C (Figure 2B). On the other hand, when the HCS concentration in the hybrid hydrogels increases, the observed thermal decomposition also slightly decreases in the temperature range 40–350°C, a consequence of the more compact structure (see below) provided by the larger number of polymeric CS chains and their electrostatic interactions with anionic HA ones (Al-Qadi et al., 2013).

Pure HA scaffolds degrade from ca. 195°C, with the first maximum at ca. 226°C and another at 240°C; meanwhile, CS shows a first broad peak at ca. 135°C, with the main decomposition step starting at ca. 245°C with a maximum at 293°C (T_m_) irrespective of the CS molecular weight, as observed from the derivative of decomposition weight loss (Figure 2B). For hybrid CS-HA scaffolds, the first two peaks at ca. 50–60°C and 135–160°C would correspond to the clearance of water present and bound to different extents to the scaffold. The true decomposition starts at ca. 185–195°C with a first small peak/shoulder at ca. 215–219°C, not visible in all scaffolds, and which would correspond to HA decomposition, and a second one at ca. 272°C corresponding to CS decomposition, respectively. The latter shifts 1–2°C to lower temperatures as the CS molecular weight decreases or the CS concentration increases (Figure 2D).

The thermal characterization was completed by means of DSC experiments (Figure 2E). According to TGA data, the thermal behavior of hybrid 3% HCS-1% HHA scaffolds is a combination of that observed for pure CS and HA ones. The thermograms show a well-defined first endothermic peak centered at ca. 77°C and a second, shallow one at ca. 224°C, and other exothermic at ca. 276°C, which is shifted regarding that of pure CS and HA scaffolds. The exothermic peak of pure HHA scaffold at ca. 217°C is completely masked probably due to the differences in concentration between HCS and HHA in the scaffold. The first endothermic peak is related to the evaporation of residual water linked to the polymeric chains, whereas the exothermic maximum is attributed to the decomposition of the polymeric chains, in correspondence with TGA data. The latter process is observed to shift to lower temperatures (ca. 266°C, respectively) when using LCS in the polymeric matrices, whereas the remaining conditions do not change within the experimental uncertainty.

**Figure 3:**
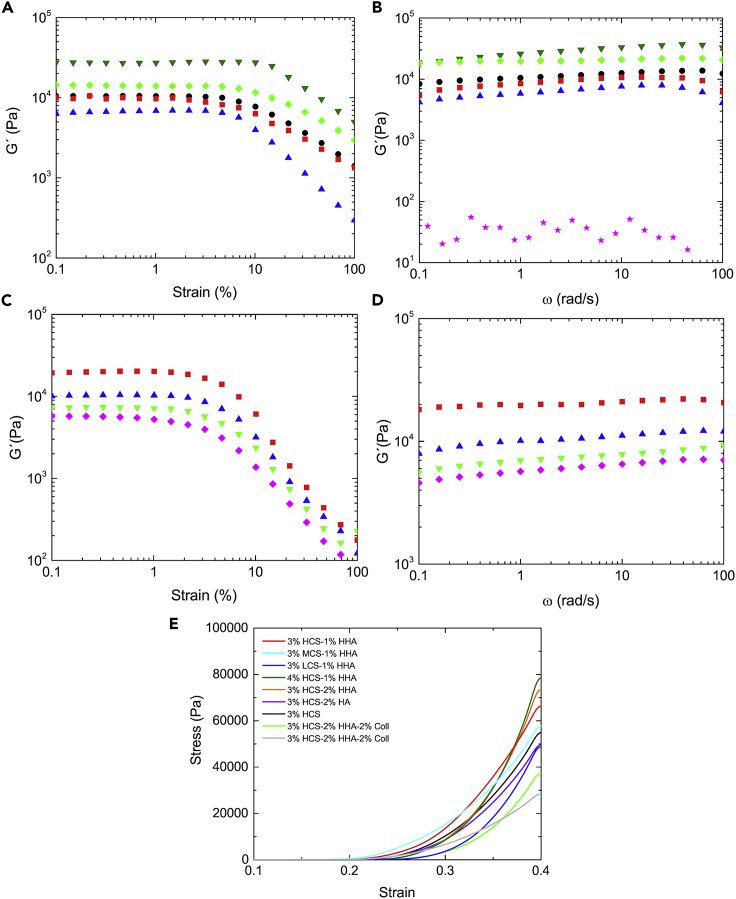
Mechanical properties Storage modulus (G′) as (A) a function of strain and (B) of frequency for 3% HCS (), 3% HCS-1% HHA (), 4% HCS-1% HHA (), 3% MCS-1% HHA (), and 3% LCS-1% HHA () hybrid scaffolds. Loss modulus (G') of 3% HCS-1% HHA scaffold (); Storage modulus (G′) as a function (C) of strain and (D) frequency for 3% HCS-2% HHA (), 3% HCS-2% LHA (), 3% MCS-2% HHA-1% Coll (), and 3% HCS-2% HHA-2% Coll () hybrid scaffolds; (E) compression tests for CS-based scaffolds of different compositions. Concentrations and compositions are indicated in the plots. Measurements were done at 37°C.

Figure 3A shows the elastic modulus (*G*′) as a function of deformation. Hybrid 3% HCS-1% HHA hydrogel shows a linear viscoelastic region (LVR) extended to strain values of ca. 5%, which is longer than that observed for pure 3% CS; conversely, the 1% HHA solution behaves as a viscous sol (not shown). The observed trend agrees with a higher crosslinked density by means of electrostatic complexation between CS and HA chains, as previously mentioned. The use of CS of lower molecular weights led to softer scaffolds with similar LVR regions as that of 3% HCS-1% HHA hydrogel; in contrast, an increase in the CS concentration in the matrix composition involved harder scaffolds with relatively longer LVR, which agrees with their larger density. It is also worth mentioning that for pure CS and hybrid CS-HA hydrogels *G*′ is much larger than *G*″ (only shown for 3% HCS-1% HHA for clarity) in agreement with gel formation and structuration upon polymer self-assembly.

Figure 3B shows that *G*′ hardly increases as a function of frequency except at the largest values where a small decrease takes place as a consequence of scaffold's structure disruption. Hybrid 3% XCS-1% HHA hydrogels with HCS and MCS in the composition display *G*′ values much larger than those obtained for pure HHA ones (not shown), which again agrees with the idea of additional stabilization of the polymeric matrix through existing interactions between HA and CS polymeric chains. Also, the increase of the CS concentration in the matrix (e.g., 4% HCS–1% HHA) reinforces such observation. The behavior observed hence resembles that of true gels.

Data derived from compression tests (Figure 3E) indicate that, in general, hybrid 3% XCS-1% YHA gels have higher moduli (i.e., the slope of stress versus strain curve at low strain) than pure HA and CS ones in agreement with a larger cross-linking point density (i.e., swelling, see below). In all cases, and as usual for compression tests, there exists an initial linear behavior at very low strains followed by a great increase of stress as strain does. Compressive Young’s moduli (E) of fully hydrated hybrid scaffolds increases with increasing CS content from 717 kPa for 3% HCS-1% HHA to ca. 1037 kPa for 4% HCS-1% HHA scaffold, respectively, whereas the decrease in the CS molecular weight in the composition leads to scaffolds with much lower E values (see Table 2).

**Table 2:** Summary of compositions and physico-chemical properties of scaffolds

Samples	Gel fraction (%)	E (kPa)	Density (g/mL)	Porosity (%)	Pore size (μm)
3% HCS	68 ± 5	615 ± 4	0.192 ± 0.007	87 ± 3	114 ± 34
3% HCS-1% HHA	74 ± 6	717 ± 7	0.169 ± 0.004	90 ± 4	96 ± 14
3% MCS-1% HHA	73 ± 3	599 ± 9	0.167 ± 0.009	92 ± 1	88 ± 12
3% LCS-1% HHA	68 ± 2	568 ± 7	0.166 ± 0.007	96 ± 2	77 ± 15
4% HCS-1% HHA	79 ± 4	1037 ± 13	0.274 ± 0.009	86 ± 6	28 ± 11
3% HCS-2% HHA	79 ± 1	981 ± 13	0.147 ± 0.008	89 ± 3	30 ± 7
3% HCS-2% LHA	82 ± 3	538 ± 2	0.139 ± 0.003	90 ± 7	29 ± 8
3% HCS-2% HHA-1% Coll	78 ± 3	532 ± 5	0.148 ± 0.005	92 ± 6	36 ± 10
3% HCS-2% HHA-2% Coll	75 ± 6	285 ± 5	0.141 ± 0.003	95 ± 4	55 ± 9

**Figure 4:**
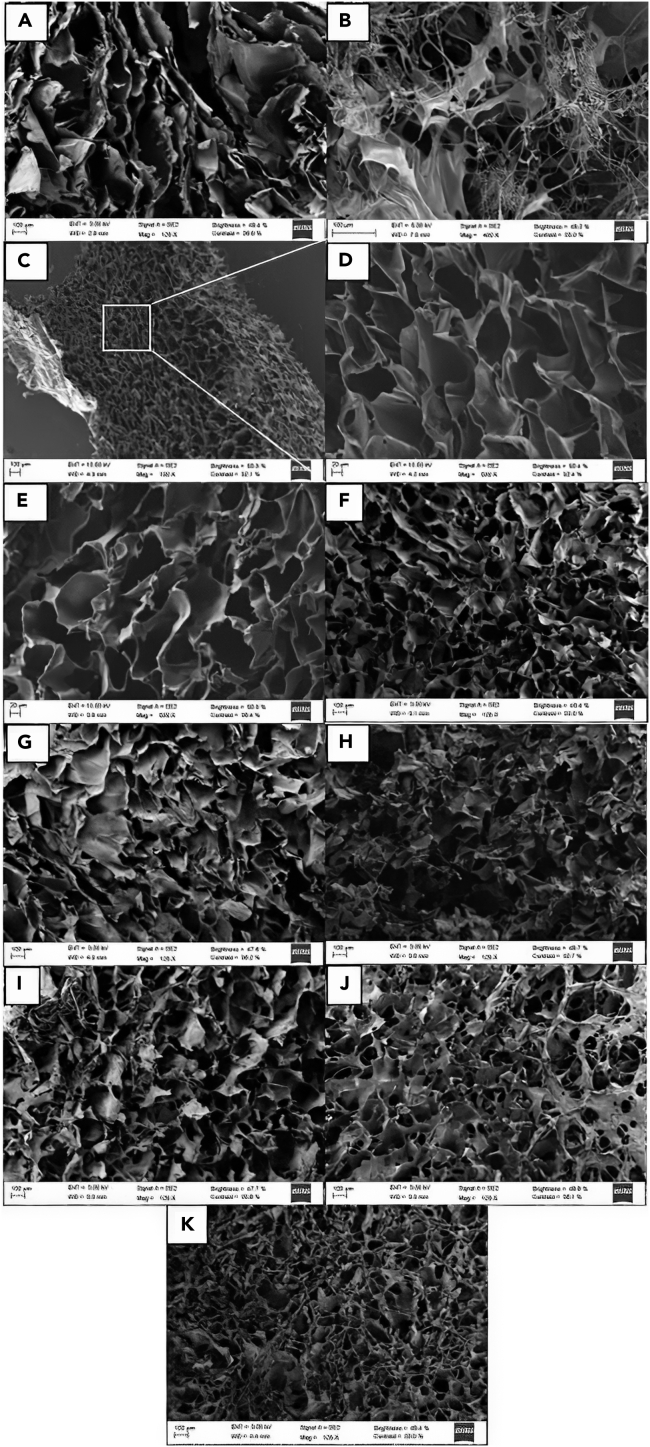
SEM images (A–K) (A) 3%HCS, (B) 1% HHA, (C) 3% HCS-1% HHA, (D) magnification of a region of 3% HCS-1% HHA, (E) 3% MCS-1% HHA, (F) 3% LCS-1% HHA, (G) 4% HCS-1% HHA, (H) 3% HCS-2% HHA, (I) 3% HCS-2% LHA, (J) 3% HCS-2% HHA-1% Coll, and (K) 3% HCS-2% HHA-2% Coll hybrid scaffolds.

In addition, it is worth mentioning that the mechanical performance of the scaffold materials is highly dependent on the porosity and pores sizes. Pores on the microporous or low mesoporous range do not have an important influence on the mechanical properties (Rege et al., 2019), whereas micropores exhibit a power-law scaling relationship (Goimil et al., 2018). Hence, scanning electron microscopy (SEM) images of the scaffold were acquired to analyze their microstructure and porosity (Figure 4).

All hybrid A% XCS-1% YHA scaffolds of different CS molecular weights and concentrations are highly porous with interconnected pores. The bulk scaffold porosity obtained by means of the liquid displacement is greater than 85% for pure 3% HCS and hybrid 3% HCS-1% HHA, 3% MCS-1% HHA, and 3% LCS-1% HHA hydrogels, confirming the presence of interconnected pores. An open, interconnected pore structure is critical for the maintenance of healthy cells *in vitro* allowing the diffusion of nutrients, oxygen, and waste materials throughout the polymeric matrix, ideal for TE applications (Kuo and Chang, 2013; Romn et al., 2011). The pore diameter associated with the different scaffold compositions was also estimated and compared by measuring the area of pore cross-sections from SEM images. The pores are not perfectly spherical, thereby precluding a direct measure of the pore cross-sectional diameter. Pure 3% HCS scaffolds show a porous laminated structure (Figure 4A), with mean pore sizes of ca. 114 μm in agreement with previous studies (Moura et al., 2011), which changes when HA is added to the composition. The hybrid 3% HCS-1% HHA scaffold has structural cues and pore sizes of ca. 96 μm (assuming mean equivalent circle diameters) and a stable cross-sectional structure (see Figure 4C) in contrast to pure HA hydrogel, which possesses a completely disorganized structure after lyophilization (Figure 4B). The former hydrogel displays a relatively regular porous structure is attributed to the phase separation of the aqueous solvent and the formed PEC complexes, followed by solvent crystal nucleation and growth during the freezing step. The open porous structure remains following sublimation during lyophilization. This structural pattern is maintained for 3% MCS-1% HHA and 3% LCS-1% HHA hydrogels, which possess pore sizes of ca. 88 and 77 μm, respectively (Figures 4D and 4E). Some small pores embedded in larger ones forming interconnections can be observed, particularly when using LCS, but their presence decreases as the CS concentration increases or CS of larger molecular mass is used. This is a consequence of the increase in the polymer solution viscosity that may hinder somehow the dendritic ice crystal formation, thereby decreasing the interconnections between adjacent walls of the pore structure, reflected in a certain decrease in scaffold porosity as observed in Table 2. The present pore sizes fall in the range of relevant values for *in vitro* cell culture (Florczyk et al., 2013, 2016; Wang et al., 2016). It is observed that the increases in the CS concentration in the hybrid scaffold to give 4% HCS-1% HHA leads to smaller pores (ca. 28 μm, Figure 4F) in agreement with the fact that hydrogels with larger and more dense polymeric contents may contain less water inside, forming smaller ice crystals during freezing and thus giving rise to smaller pore sizes after lyophilization.

The observed interconnected porosity and pore sizes were additionally confirmed by means of 3D-reconstructed images obtained by computed microtomography (μCT) for selected 3% HCS-1% HHA and 4% HCS-1% HHA scaffolds, as examples.

**Figure 5:**
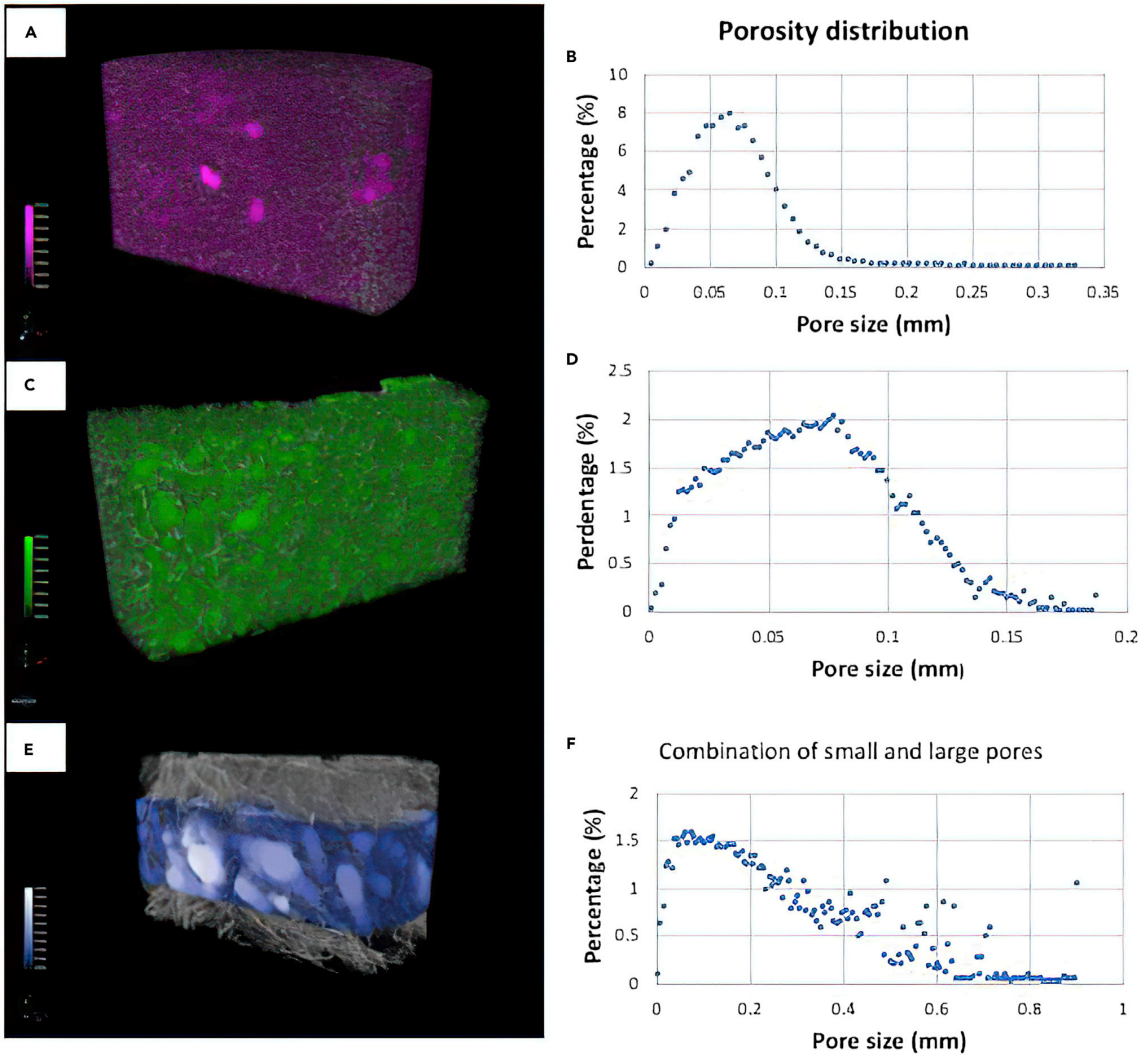
3D reconstructed μCT images and pore size distributions (A–F) (A, B) 3% HCS-1% HHA; (C, D) 4% HCS-1% HHA; and (E, F) 3% HCS-2% HHA-2% Coll, respectively.

Figure 5 confirms that 3% HCS-1% HHA and 4%- HCS-1% HHA hydrogels show porosities of ca. 90 and 88%, respectively, in great agreement with data obtained by the liquid displacement method. Pore distributions confirm the microscopic character of the pores with mean sizes of ca. 75 and 63 μm, in relatively fair agreement with values measured from SEM images. It is also confirmed that porosity decreases as the CS polymer content increases within the experimental uncertainty as a consequence of providing thicker pore walls and fewer pore interconnections.

**Figure 6:**
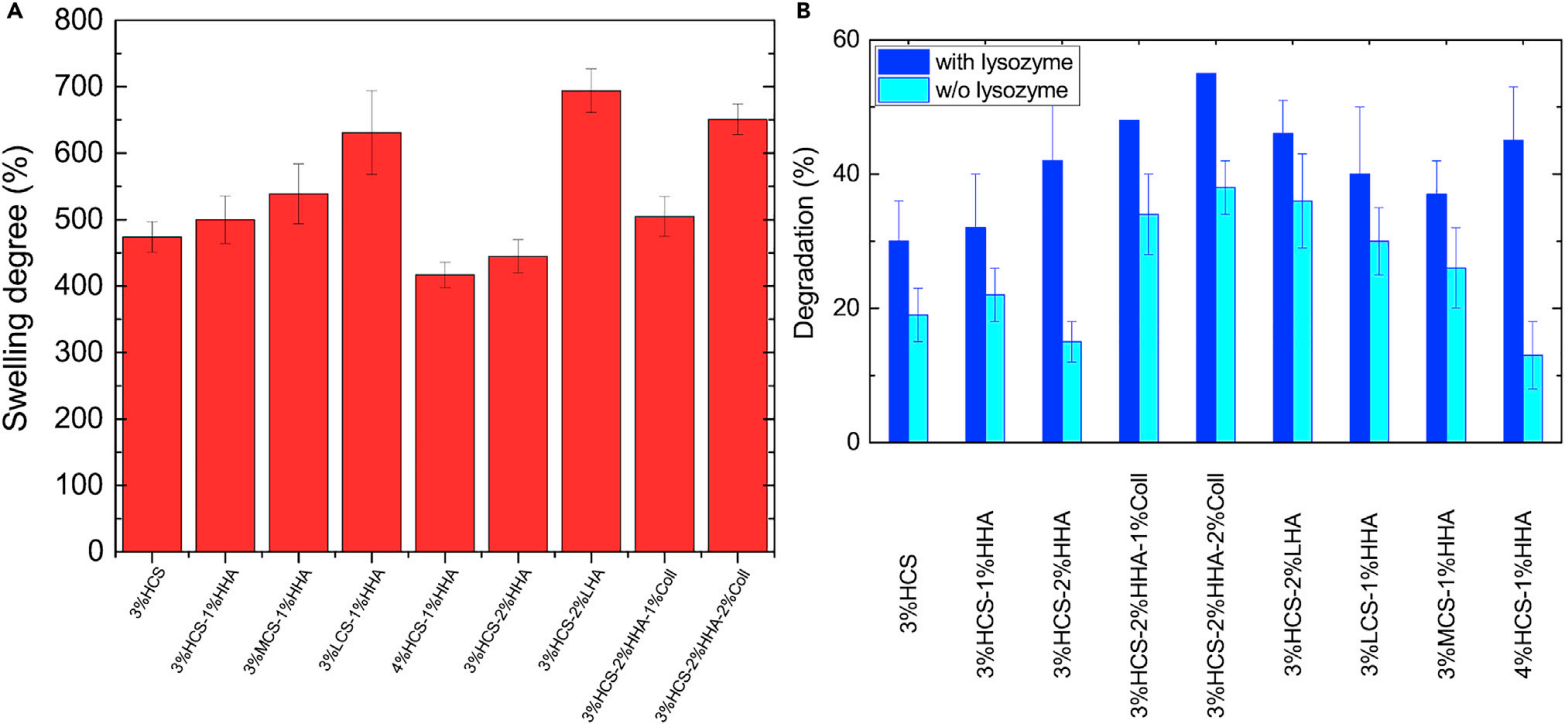
Swelling degree at equilibrium and degradation (A and B) (A) Swelling degree at equilibrium for different CS-based hydrogels and (B) degradation of hybrid hydrogels after 28 h in the presence and absence of lysozyme.

On the other hand, the swelling capacity of the hydrogels decreases as the total polymer content increases, i.e., the porosity was tunable by changing the polymeric concentration (see below) (Gutiérrez et al., 2007; Kuo and Chang, 2013) in agreement with structural information derived from SEM and μCT images. In this regard, the hybrid 3% HCS-1% HHA scaffold possesses a swelling capability of ca. 500%, which is slightly larger than that of pure 3%HCS (474%). Pure 1% HHA scaffold was unable to retain water due to its soft gel characteristics (G'> G″ and G'< 50 Pa). If the CS concentration increases in the hybrid scaffold, the swelling capability is slightly lower; ca. 417% for the 4% HCS-1% HHA hydrogel scaffold related to a decrease in the hydrogel porosity as well as an increase in the crosslinking density between HA and CS through electrostatic interactions, hydrogen bonding and/or hydrophobic interactions, as mentioned previously, which, in turn, diminishes the hydrogel flexibility and expansion capacity. Conversely, the use of MCS and LCS to construct the scaffolds favors swelling with values at the equilibrium of 539 and 631%, respectively, for hybrid 3% MCS-1% HHA scaffolds and 3% LCS-1% HHA, respectively (Figure 6A). The water absorption capacity is attributed to both the hydrophilic moieties present in HA and CS chains and the structure of the hydrogel scaffolds, whereas the existing dissolution resistance originates from the ionic crosslinking of the polymeric chains.

To evaluate the biodegradability of the prepared hydrogel scaffolds, these were immersed in PBS buffer containing the protein lysozyme. Figure 6B shows the extent of degradation of the polymeric scaffolds after 28 days of incubation in the presence of the enzyme. Control experiments in the absence of the protein were also performed to decouple enzymatic degradation from network erosion or dissolution within the time frame of the experiments. The degradation shown by the hybrid 3% HCS-1% HHA scaffold was ca. 35%, slightly better than pure 3% CS; this is attributed to the formation of stronger ionic bonds, that is complexation, between CS and HA; conversely, nonenzymatic degradation of the hybrid scaffold was only ca. 21%, confirming the bioactivity of lysozyme. Moreover, both the increase in CS concentration and the use of CS of lower molecular weights in the hybrid scaffolds lead to larger degradation upon incubation with the protein (ca. 44% and 38%–40%, respectively).

#### Effect of HA concentration and molecular weight

The increase in HA concentration in the hybrid CS-HA scaffolds leads to subtle changes in the physico-chemical and structural properties of the hybrid hydrogels. Figure 1 shows the FTIR spectra of 3%HCS-1%HHA and 3%HCS-2%HHA where scarce changes were observed, hardly any enhancement of bands at ca. 1323 cm^−1^ and 1640 cm^−1^ and the appearance of a shoulder at 990 cm^−1^. The incorporation of LHA in the composition does not involve any relevant modification in the resulting FTIR spectrum of the 3% HCS-2% LHA hybrid hydrogel.

Regarding the thermal properties, the increase of concentration HHA in the hybrid scaffold composition to give 3% HCS-2% HHA leads to a visible increase in the peak height observed at ca. 212–214°C corresponding to the HA degradation, which is shifted to lower temperatures compared with 3% HCS-1% HHA scaffolds. In addition, the degradation peak of HCS remained invariable at ca. 271–274°C (Figure 2E). Moreover, both 3% HCS-2% HHA and 3% HCS-2% LHA hybrid scaffolds reach total weight losses of ca. 56% at 350°C and of ca. 66% at 600°C, respectively (Figure 2D). As observed, residual masses are lower than those observed for 3% XCS-1% HHA gels due to the presence of a high quantity of HA in the formulation that degrades at lower temperatures than CS. From DSC data, the first endothermic maximum shifts to higher temperatures (ca. 80–81°C), whereas the opposite behavior is observed for the exothermic one is (261°C) in agreement with the increase in HA concentration for 3% HCS-2% HHA hybrid scaffolds (Figure 2F). In addition, a small, broad exothermic peak at 227°C can be observed for the 3%HCS-2%HHA scaffold, which should correspond to the degradation peak of HHA and which now can be observed as a consequence of the concentration increase of HA. However, a shift of the main exothermic peak of CS to ca. 257°C masked the former when using a low-molecular-weight HA, 3% HCS-2% LHA.

Figure 3C shows that hybrid 3% HCS-2% HHA and 3% HCS-2% LHA scaffolds have an LVR up to 1.5% strain, which is slightly small than that of 3% HCS-1% HHA. However, the 3% HCS-2% HHA hydrogel is stiffer than the 3% HCS-1% HHA one probably due to an enhancement of ionic interactions between HA and CS as well as the increase in the total polymeric content. Conversely, the use of LHA in the 3%HCS-2%LHA hydrogel gives rise to softer scaffolds with *G′* values slightly smaller than that of 3%HCS-1%HHA. This results from the lower viscosity of LHA despite the larger total polymeric content. In addition, 3% HCS-2% HHA scaffold shows a frequency-independent behavior typical of true gels, whereas 3% HCS-2% LHA one displays a weak *G′* frequency dependence (see Figure 3D) as for weak gels of lamellar liquid crystals formed by double tails ionic surfactants, i.e., aerosol OT/water and didodecyldimethylammonium bromide (Alcantara and Vanin, 1995; Soltero et al., 2000). Regarding the resistance to compression, 3% HCS-2% HHA and 3% HCS-2% LHA hydrogels showed elastic moduli of 981 and 538 kPa (see Table 1), respectively. The larger modulus of the formed hydrogel confirms its resistance derived from ionic crosslinking between CS and HA, in agreement with rheology data.

The modification of either the HA concentration or molecular weight is also reflected in structural data. For example, the increase in HA concentration in the scaffolds leads to an increase in the gel fraction (79%) result of the enhancement of ionic interactions between components, whereas both the density and porosity of the hydrogels slightly decrease due to the lower density of HA (Table 2). SEM images confirmed that an increase in the HA weight percentage in the scaffolds gives rise to more rugged void walls for the hybrid 3% HCS-2% HHA scaffold than those observed for the hybrid 3% HCS-1% HHA hydrogel, that is, a very porous structure but with more disorganized pores of mean sizes of ca. 30 μm is still retained (Figure 4G), in agreement with the structure observed in other hybrid hydrogel scaffolds containing HA (Chang et al., 2013; Gilarska et al., 2018; Velasco- Rodriguez et al., 2021). Conversely, the use of LHA in the scaffold composition does not seem to have a great impact on the scaffold structure. Slight increases in both gel fraction (82%) and porosity (ca. 90%) are observed as well as lower density due to the reduction of HA viscosity in the polymeric mixture, showing a structure characterized by the observation of nonregular pores of mean size of ca. 29 μm, which are interconnected in different areas by somehow loosely bound elongated polymeric material to larger extents (Figure 4H).

Hybrid 3% HCS-2% HHA scaffolds were able to retain less water inside compared with 3%HCS-1%HHA ones with up to ca. 445%, which further increases up to ca. 694% when using LHA in the formulation instead (Figure 6A). As LHA is more hydrophilic and much less viscous than HCS, it may create more void volumes within the gel structure due to the irregular arrangement of the chains in the gel, that is, HA chains would intermingle with CS ones to form a hybridized matrix able to absorb more water and, as a result, the size of ice crystals expanded during freezing yielded relatively larger voids.

In addition, the enzymatic degradation extents shown by hybrid 3% HCS-2% HHA and 3% HCS-2% LHA scaffolds in the presence of lysozyme were ca. 42% and 45%, respectively, similar to those of the other scaffold compositions containing less HA. However, it is necessary to highlight the lower resistance to dissolution of the 3%HCS-2%LHA scaffold, which showed degradation of ca. 35% in the absence of the protein, much higher than that of the 3% HCS-2% HHA counterpart, ca. 19% (Figure 6B).

#### Effect of collagen incorporation and concentration

As mentioned previously, the addition of Coll to the scaffolds serves to provide additional cell adhesion motifs and high tensile strength to favor cell adhesion and proliferation; moreover, this protein can adopt different structures similar to biological ones, which contribute to its high level of biocompatibility and biodegradability. When collagen is introduced to obtain, for example, 3% HCS-2% HHA-1% Coll and 3% HCS-2% HHA-2% Coll hybrid scaffolds, the characteristic bands of this protein such as amide A, amide B, amide I, amide II, and amide III are overlapped with those corresponding to both CS and HA (Figure 1A). However, a slight increase in intensity can be observed for amide I and amide II bands, at ca. 1637 and 1555 cm^−1^; in addition, asymmetric stretching of the C-*O*-C bridge and C-O vibration at ca. 1063 and 1027 cm^−1^, respectively, enhance as the collagen concentration in the hydrogel composition increases, hence, denoting the incorporation of the protein. The incorporation of Coll and its distribution along the scaffold could be additionally confirmed by Raman spectroscopy. Figure 1C shows that Coll is satisfactorily incorporated within the polymeric matrix, and it is well dispersed along the whole scaffold without signals of phase separation even as its concentration increases.

Regarding the thermal characteristics, the incorporation of Coll leads to a certain shift of the maximum located at ca. 271°C to ca. 280°C for 3% HCS-2% HHA hydrogels and 3% HCS-2% HHA-2% Coll hydrogels, respectively, in agreement with the higher degradation temperature of Coll (close to ca. 300°C) (Sorushanova et al., 2019). The incorporation does not involve significant changes in the degradation extents observed at 350°C and 600°C when compared with those of 3% HCS-2% HHA and 3% HCS-2% LHA ones, being again ca. 55%–56% and 66%–68% for 3% HCS-2% HHA-1% Coll and 3% HCS-2% HHA-2% Coll hydrogels, respectively (Figure 2D). In DSC plots, the presence of Coll might be denoted by the broadening and shift of the exothermic peak to larger temperatures ca. 275°C, confirming the presence of this protein if compared with 3% HCS-2% HHA scaffolds, which show the maximum of this peak at ca. 261°C (Figure 2F). In addition, for 3% HCS-2% HHA-1% Coll and 3% HCS-2% HHA-2% Coll scaffolds, the first sharp endothermic maximum shifts to ca. 78–80°C compared with 3% HCS-1% HHA, 3% HCS-2% HHA, and 3% HCS-2% LHA scaffolds, possibly originated from the existence of some humidity in the sample. The exothermic peaks corresponding to HA and CS degradation are maintained at ca. 260–262°C but are overlapped and shifted to larger temperatures as the collagen concentration increases due to the larger degradation temperature of this protein (Lewandowska et al., 2016). Also, a shallow minimum observed at ca. 208°C may be indicative of the glass transition (T_g_) of the protein (Gilarska et al., 2018).

The incorporation of Coll in the hybrid scaffolds also leads to subtle changes in their rheological behavior. The presence of this protein within the scaffold compositions involves a progressive decrease in *G′* values as the collagen concentration increases as well as a reduction in the LVR (up to ca. 1%), as observed when comparing 3% HCS-2% HHA, 3% HCS-2% HHA-1% Coll and 3% HCS-2% HHA-2% Coll (Figure 3C). These observations agree with previous reports (Pietrucha, 2005) in which the presence of Coll in the formulation worsens the rheological properties, that is, allows the development of softer hydrogel scaffolds due to the poor mechanical properties of this protein. Compression test data also agree with the former observation, that is, relatively lower Young's moduli are obtained as the collagen concentration in the hybrid scaffolds increases (see Table 2). In addition, the presence of Coll in the scaffolds also involves changes in their structure. Gel fractions and porosities of Coll-containing scaffolds slightly increases as the concentration increases, whereas the scaffold density becomes slightly larger due to the presence of more material in the composition. Moreover, the introduction of Coll leads to scaffolds with apparently more disorganized and irregular structures with porosities of ca. 90% and pore sizes of ca. 36 and 55 μm for 3% HCS-2% HHA-1% Coll and 3% HCS-2% HHA-2% Coll scaffolds, respectively (Figures 4I–4K). Figures 5E and 5F shows a 3D reconstructed image from μCT experiments of a 3% HCS-2% HHA-2% Coll scaffold, which confirms the former observations. In this way, a loose skeleton composed of elongated crosslinked structures, also visible in SEM images, are noted, with a derived porosity of ca. 95% and two populations of pores with different sizes: one well below 70 μm, in agreement with images from SEM, and another one between 200 and 600 μm. This structure involved a slightly larger swelling capacity of Coll-containing scaffolds, particularly for 3% HCS-2% HHA-2% Coll one (ca. 651%, see Figure 6A), and lower resistance to enzymatic attack, with biodegradation extents of ca. 48% and 55% after 28 days of incubation for 3% HCS-2% HHA-1% Coll and 3% HCS-2% HHA-2% Coll hydrogels, respectively (Figure 6B), which is significantly larger than those obtained for 3% HCS-2% HHA and 3% HCS-1% HHA counterparts.

In summary, the rheological and mechanical properties are essential for the intended biological functions and mechanical behavior of the designed scaffolds. These characteristics are primarily dependent on the hierarchical organization of polymeric chains within the scaffolds and their mutual interactions, porosity and pore sizes of the developed structure, etc. In this manner, different parameters can be tuned (e.g., polymeric total content and mixing ratios, the extent of crosslinking, temperature, pH, etc.) to achieve the scaffold structure with the desired viscoelastic and mechanical properties. For example, scaffolds useful for brain and nerve regeneration must have storage moduli in the range 1 · 10^2^–1·10^3^ Pa, whereas those for substituting liver, cartilage, relaxed muscle, breast, or glad tissues should range between 1 × 10^3^ Pa and 5 × 10^4^ Pa or for bone regeneration, which should be above 10^5^ Pa. In the same way, scaffolds for bone and cartilage engineering must possess very high Young's moduli (>1 × 10^4^Pa), whereas those devoted to the spinal cord, nerve, and brain tissues should be rather low. In addition to mechanical and rheological properties, porosity also plays a key role. In this way, scaffolds with porosities above 70% are ideal for tissue engineering purposes, as they should allow a correct permeation of nutrients and facilitate cell colonization and growth (Velasco- Rodriguez et al., 2021). Here, a part of the developed scaffolds presented may resemble the adhesive and rheological properties of skin tissue at different body sites, that is, Young's and elastic moduli of ca. 0.3 to 1.2 MPa and ca. 10^4^–10^7^ Pa) (Griffin et al., 2017), respectively, thus becoming optimal matrices for intended tissue engineering applications as well as transdermal drug delivery depots. In this regard, from the perspective of skin tissue regeneration, the scaffold should act as a moist bed with a proper hydrophilic-hydrophobic balance that in turn would support growth, migration, and proliferation of the skin cells to heal up the deceased tissue.

### Biological evaluation

**Figure 7:**
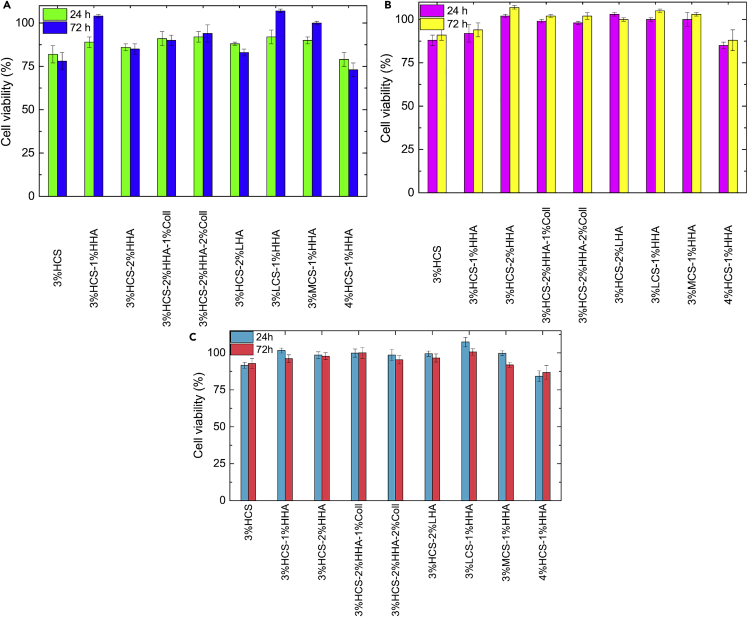
Cytotoxicity of different CS-based hybrid scaffolds after 24 and 72 h (A–C) (A) BALB-3T3 mouse fibroblasts, (B) HaCaT human keratinocytes, and (C) MSC human adipose stem.

Once the different types of hybrid scaffolds were physico-chemically characterized, their biological evaluation *in vitro* was conducted. As commented previously, HA and Coll are the main components of the extracellular matrix, for example, in skin tissue (Papakonstantinou et al., 2012), whereas CS has shown outstanding cell adhesion as well as excellent mechanical properties when used as a constituent of polymeric matrices (Huang et al., 2019). To this end, mouse Balb-3T3 fibroblasts and human keratinocytes were chosen as model cell lines for being cultured with the scaffolds to evaluate their potential biocompatibility for prospective applications as intradermal drug depots and/or biomaterials for filling intradermal voids and subsequently favored skin ECM repair. To do that, two different cytocompatibility experiments were done. In the first one, the scaffolds were incubated for 96 h in culture medium, and the resulting lixiviates were added to cultured cells and incubated for another 24 and 72 h. Figure 7 shows that all hybrid scaffolds were cytocompatible, with viabilities ranging from 80% to 95% and 73% to 107%, ca. 85% to 103% and 88% to 107% and ca. 84% to 104% and 85% to 99% at 24 and 72 h for mouse fibroblasts, human keratinocytes, and mesenchymal stem cells (MSCs), respectively. Fibroblasts display a slightly lower average viability probably due to the sensitivity of this cell line to the surrounding environmental conditions, which makes it a typical control cell line for testing the biocompatibility of a vast range of materials (Yassue-Cordeiro et al., 2019). Moreover, no significant differences were observed in cell viabilities between the different scaffolds in relation to the polymer composition and concentration but scaffolds, particularly for keratinocytes and MSCs, where CS is the main component that seems to be slightly more toxic to cells probably due to some excess of cationic charges interacting with the cellular membranes. It is also worth mentioning that scaffolds containing HA and Coll in the composition are slightly more cytocompatible within the experimental uncertainty, probably as a consequence of scaffold structure and composition, the latter resembling, for example, that of skin ECM.

**Figure 8:**
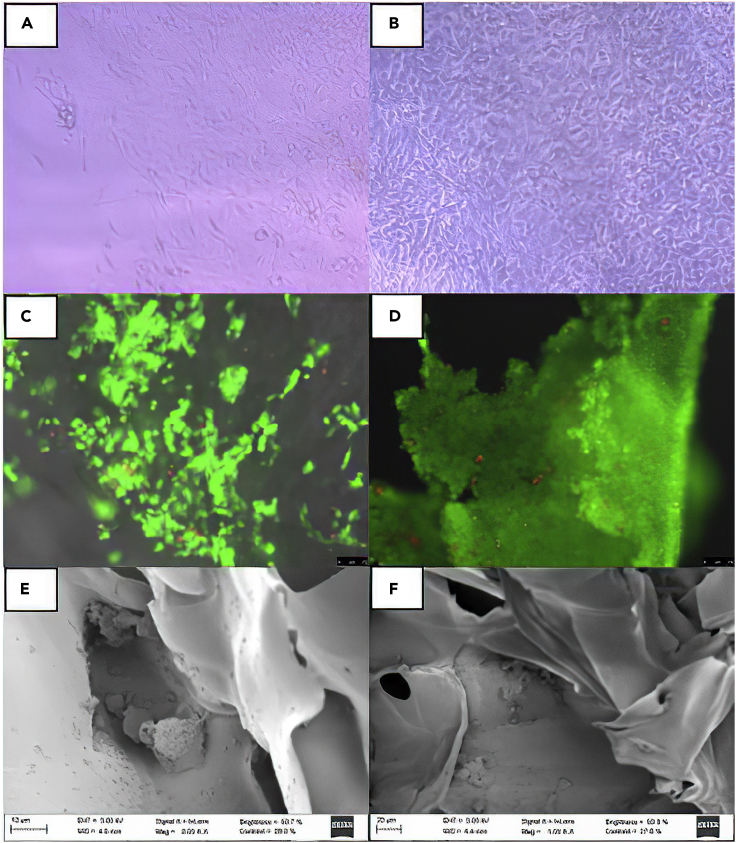
Cell viability and proliferation in the scaffolds (A–D) (A) and (B) Optical microscopy images of proliferation and (C) and (D) epifluorescence images from Live/Dead assay illustrating the proliferation of BALB 3T3 mouse fibroblasts and human HaCaT keratinocytes after 3 and 7 days on 3%HCS-1%HHA-1% Coll as an example. (E) and (F) SEM images depicting the infiltration of HaCaT cells within a 3% HCS-1% HHA-1% Coll hybrid scaffold.

Cell viability and proliferation in the scaffolds were also analyzed by optical and fluorescence microscopy and SEM. Figures 8A and 8B shows that, for example, fibroblast cells were able to adhere to the surface of the hybrid scaffolds. In addition, calcein AM-propidium iodide staining confirms that keratinocyte cells remain viable (Figures 8C and 8D). SEM images also confirmed that cells can infiltrate through the porous polymeric matrices and adhere and proliferate within the pores, as observed for keratinocytes (Figure 8E) that display their classic round shape when harvested onto nonplanar surfaces. Further incubation leads to a large spreading of these cells inside and on top of the scaffolds, as confirmed in Figures 8D–8F. Hence, the proliferation and infiltration of cells within the present scaffolds as well as their reasonable hierarchical structure, suitable and tunable physico-chemical properties (porosity, viscoelastic, mechanical, etc.), and adhesiveness make these polymeric matrices optimal for intended tissue engineering applications; in particular, the tunable rheological and mechanical properties as well the structure as a function of polymeric composition and concentration would make these structures interesting for intended skin tissue regeneration, and/or a transdermal drug delivery depots, as well as 3D *in vitro* models reproducing the complexity of natural skin to allow mechanistic studies to decipher and understand the underlying mechanisms of endothelium inflammation and plaque formation and to validate the utility and therapeutic gains of incorporated drugs and drug-loaded nanocarriers in skin regeneration. However, some additional *in vitro* experiments are indeed required to confirm that the present scaffolds can support the complex organization of cells mimicking complex tissue such as skin, with further corroboration *in vivo* animal models. Here, an important key to achieving not only the cell-tissue organization but also suitable vascularization would be of great importance. In this regard, most of the present scaffolds looking for regeneration or drug depot applications would involve a surgery step to be implanted in the animal body site. Hence, the search for a responsive hydrogel scaffold based on the present polymers to get an *in situ* gelling matrix by incorporation of an additional thermal-/pH-sensitive polymer component would be an additional advantage.

### Limitations of the study

The obtained hybrid scaffolds showed good characteristics for several potential tissue engineering applications such as skin regeneration. Nevertheless, it is necessary to consider that only a range of concentrations was tested according to the expected structural and rheological properties. Also, *in vitro* experiments were limited to tests evaluating the hydrogels' cytocompatibility and capability to sustain cell proliferation and infiltration inside the scaffolds. Additional studies would be required to analyze if the present hybrid scaffolds biologically meet the requirements as matrices for successful skin tissue regeneration, for example, including the controlled delivery of differentiating factors or maintenance of biologically active compounds inside to control and enhance cell proliferation, differentiation, and structuration. In this line, additional *in vivo* studies should be performed to confirm the efficacy of the developed matrices as suitable synthetic ECMs with suitable vascularization to support structured cell tissue. Nonetheless, the present work focused not only on the development and extensive characterization of biocompatible hydrogel matrices based on biopolymers but also on the establishment of suitable relationships between scaffolds composition and concentration with architecture and physical properties.

## STAR★Methods

### Key resources table

**Table:** 

REAGENT or RESOURCE	SOURCE	IDENTIFIER
**Chemicals, peptides, and recombinant proteins**		

Chitosan low molecular weight	Sigma-Aldrich	CAS: 9012-76-4
Chitosan medium molecular weight	Sigma-Aldrich	CAS: 9012-76-4
Chitosan high molecular weight	Sigma-Aldrich	CAS: 9012-76-4
Collagen type I	EMD Millipore Corporation	Catalog: 08-115 Lot: 2812057
Phosphate buffered saline	Sigma-Aldrich	MDL number: MFCD00131855
Ammonium hydroxide	Sigma-Aldrich	CAS: 1336-21-6
Lysozyme from chicken egg	Sigma-Aldrich	CAS: 12650-88-3
Acetic acid	Sigma-Aldrich	CAS: 64-19-7
Cell counting Kit −8 (CCK-8)	Dojindo Molecular Technologies, Inc.	Code: CK04
Hyaluronic acid sodium salt -Average MW 80,000–100,000 Da	Carbosynth.	Batch number: FH634271601
Ethanol		CAS: 64-17-5

**Experimental models: Cell lines**		

Mouse Balb/3T3	ATCC	CCL-163
Immortalized human dermal keratinocytes (HaCaT)	Merck	102-05A
StemPro® Human Adipose-derived Stem Cells	Gibco	R7788115

### Resource availability

#### Lead contact

Further information and requests for resources and reagents should be directed to and will be fulfilled by the lead contact, Pablo Taboada (pablo.taboada@usc.es) and Morteza Mahmoudi (mahmou22@msu.edu).

#### Materials availability

All materials are from commercial sources and are widely available*.*

#### Data and code availability

Original data are available from corresponding authors and stored in the research group web servers fulfilling all data management requirements. This paper does not report the original code. Any additional information required to reanalyze the data reported in this paper is available from the lead contact upon request.

### Experimental model and subject details

#### Cell lines

Mouse Balb/3T3 cell fibroblast from mouse embryos and immortalized human dermal keratinocytes (HaCaT) cell lines were purchased from Sigma Aldrich and grown under standard culture conditions (*i.e.* in a humidified atmosphere with 5% CO_2_ at 37°C) in DMEM supplemented with 10% FBS, 0.1 mM NEAA, 2 mM L-Glutamine, 1 mM sodium pyruvate, and 1% penicillin-streptomycin, respectively.

The mesenchymal stem cells (MSC) isolated from human adipose tissue supplied by Gibco were grown in MesenPRO RS^TM^ supplemented with 2% growth supplement and 1% of 2 mM L-Glutamine, in a humidified atmosphere with 5% CO_2_ at 37°C.

Cells were passaged until reaching 75% optical confluence. Confluent monolayers were then treated with the trypsin-EDTA solution and incubated for 4 min under culture conditions for detachment. Cells were then pelleted, resuspended in culture medium, and seeded onto scaffolds and tissue culture plates (TCP) as required for the assay.

### Method details

#### Scaffold preparation

Pure and hybrid hydrogels of different compositions were prepared by means of a polyionic self-assembly process (see Table 1). Pure CS hydrogels were formed by dissolving CS into a solution of water- 1% (v/w) acetic acid (AA) under stirring at 300 rpm. Then, this solution was kept at the steady-state condition at room temperature for 1 h to remove potential bubbles. To form hybrid A%XCS-B%YHA hydrogels (where A, B % denote the wt% concentration of CS and HA in the scaffolds, whereas X and Y denote the molecular weight of CS and HA, respectively, H = high molecular weight; M = medium molecular weight; L = Low molecular weight) an equal volume of HA at suitable concentration was mixed with the CS solution drop by drop at room temperature and the solution mixed at 300 rpm for 24 h. To form A%XCS-B%YHA-C%Coll hybrid scaffolds (where C% denote the wt% concentration of Coll in the scaffolds) a suitable volume of Coll solution in PBS buffer was added to the former mixed CS-HA solution and stirred overnight. The final polymer solutions were placed into 24 well plates, frozen at −20°C for 24 h and subsequently lyophilized for an additional 72 h. Prepared scaffolds were sectioned into 5 mm thick discs, neutralized in 25% (v/v) ammonium hydroxide for 1 h under vacuum and washed by deionized water 4 times and soaked in PBS pH 7.4 overnight to neutralize and remove the excess base. Then, scaffolds were again frozen at −20°C and lyophilized. Finally, the polymeric matrices were sterilized with 70% (v/v) ethanol for 1 h and placed in an orbital shaker overnight to eliminate excess ethanol. All scaffolds were stored in the freezer under sterile conditions before being used for biological response evaluation.

#### Gel fraction

The gel percent of the hydrogels was determined gravimetrically. The different lyophilized scaffolds were previously weighted and subsequently immersed in 20 mL of 1X PBS for 8 days at room temperature and freeze-dried. The gel percent was calculated by the following equation:

(Equation 1) $$Gel\;\left(\%\right)=100\times \frac{W_{w}}{W_{i}}$$

where *W*_*i*_ and *W*_*w*_ are the weights (g) of the hydrogel before and after washing to extract the soluble parts. All the experiments were performed by triplicate.

#### Apparent density

The apparent density of scaffolds was evaluated from the weight to volume ratio of the lyophilized hydrogel. The apparent density, ρ, was obtained from the following equation:

(Equation 2) $$\rho =\frac{w}{\pi \times {\left(D/2\right)}^{2}\times H}$$

where ρ is the apparent density (g/cm^3^), *w* is the weight of the hydrogel (g), *D* the diameter (cm), and *H* the thickness of the hydrogel (cm), respectively.

#### Porosity

To determine the porosity of the scaffolds a liquid displacement method was used (Guan et al., 2005). The scaffolds were immersed into a graduated cylinder containing a known volume (*V*_*1*_) of ethanol since this is a non-solvent for polymers that penetrates inside the pores quickly without inducing dissolution, shrinkage or swelling. Then, the cylinder was placed under vacuum to force the ethanol into the pores of the scaffold until no air bubbles are observed emerging from the sample. The total volume of ethanol and scaffold was considered *V*_*2*_. The scaffold was finally removed from ethanol and the remaining ethanol volume was measured as *V*_*3*_. The porosity (P) of the scaffold was evaluated as:

(Equation 3) $$P\left(\%\right)=\frac{V_{1}-V_{3}}{V_{2}-V_{3}}\times 100$$

Experiments were carried out in triplicate.

#### Fourier transform infrared spectroscopy

The chemical characterization of the scaffolds was done by attenuated-reflectance Fourier transform infrared spectroscopy (ATR-FTIR). Dried scaffolds were placed on a micro-sample cup. Data acquisition was performed using an FT-IR spectrometer (Varian 670, Agilent, Santa Clara, CA, USA) coupled to a mapping microscope (Varian 620-IR, Agilent, Santa Clara, CA, USA) and an ATR diamond accessory. The samples were analyzed in the interval from 400 to 4000 cm^−1^ with a spectral resolution of 4 cm^−1^ and 64 scans min^−1^ for a total of 100 scans for each spectrum.

#### Raman imaging

Raman spectroscopy data of pure (controls) and hybrid hydrogels were obtained with a WITec Confocal Raman microscope Alpha 300R+ (Ulm, Germany). Dried hydrogel samples were homogenized with PBS to avoid potential background fluorescence. Surface and in-depth distribution of the two different polymers inside hybrid hydrogels were determined using a frequency-doubled laser at 532 nm at an output power of 7 mW and 600 mm grating. Raman spectra for image compositions were recorded using a 100X Zeiss, EC Epiplan-Neofluar DIC objective (Oberkochen, Germany) with a numeric aperture of 0.9. Image resolution was set at 1024 × 127 active pixels, with a total of 22,500 spectra per image at a scan speed of 20 s per line and an integration time per pixel of 0.13 s. Data acquisition was driven by the WITec Control software (Ulm, Germany). Peak identification in hybrid hydrogels was recorded and compared.

#### Thermal analysis

The thermal behavior of the scaffolds was analyzed by thermo-gravimetrical analysis (TGA) and differential scanning calorimetry (DSC). The decomposition behavior and thermal stability were analyzed using a TGA 55 thermogravimeter analyzer (TA Instruments, USA). 5 mg of scaffold samples were heated in a platinum pan from 0 to 700°C at 10°C/min under N_2_ atmosphere. DSC data were acquired using a Q-100 differential scanning microcalorimeter (TA Instruments, USA). After calibration with indium and lead standards, 3 to 5 mg of the scaffold was placed into hermetic aluminum pans (900793.901) and heated from 0 to 300°C at a heating ramp of 10°C/min under N_2_ atmosphere. All tests were replicated at least 3 times.

#### Oscillatory rheological measurements

The rheological properties of the scaffolds were determined using an Anton-Paar MCR301 constant stress rheometer with a 15 mm parallel-plate geometry (lower glass plate) and a gap of 240 mm. An evaporation blocker stage was used along the experiments to avoid sample dehydration. Firstly, oscillatory strain sweep measurements were performed to obtain the linear viscoelastic region (LVR) using a deformation range from 0.1 to 100% at a frequency (ω) of 6.28 rad/s (1 Hz) and 37°C. The LVR region is defined as the deformation range where the elastic modulus (*G*′) is independent of the applied deformation (%*γ*). Once the LVR was determined, changes in the mechanical properties as a function of time were monitored for a set of scaffolds by means of amplitude (0.1–100% at 10 rad/s) and frequency sweep experiments (0.1–100 rad/s at a constant strain of 0.1%) within the LVR at 37°C.

#### Mechanical compression tests

Scaffolds were subjected to unidirectional compression tests in a tensile bench with a 30 kg load cell (TA.TX*Plus*, Stable Micro Systems, Ltd., Godalming, UK) at a cross-head speed of 1 mm/min. All the experiments were performed at room temperature (25°C), atmospheric pressure and 45% relative humidity. Young's modulus (E) was calculated from the slope in the linear section of the stress-strain curve. Three replicates were used for each hydrogel composition.

#### Field-emission scanning electron microscopy

The morphology and microstructure of the scaffolds were evaluated using field-emission scanning electron microscopy (FESEM, Zeiss Ultra-Plus, Germany). Dried scaffolds were fixed with conductive adhesive on aluminum supports, sputter-coated with iridium, and observed at an accelerating voltage of 20 kV. ImageJ software was used to analyze pore size, shape and distribution.

#### Computed microtomography

The porosity and pore size distribution of selected scaffolds were analyzed by computed microtomography using a Skyscan 1272 instrument (Bruker, Germany) operated at 40 kV with a voxel resolution of ca. 3 mm and 2k resolution. 3D images were reconstructed and analyzed 3D suite software package (Bruker, Germany). Firstly, a region of interest (ROI) of ca. 4 ′ 4 ′ 4 mm^3^ and representative of the whole scaffold was isolated to evaluate the influence of the working parameters on the resulting data. Afterwards, the calibration of the thresholding of the grey-scale was performed to differentiate the void fraction (pores and interconnections) from the solid material. Once done, the porosity of the scaffolds was calculated in the ROI expressed as a percentage of void voxels to the total number of voxels by means of CTAn and CTVox routines of the 3D Suite software package.

#### Swelling

The swelling behavior of the scaffolds was measured gravimetrically. Pure and hybrid dried scaffolds were submerged in 10 mL of 1X PBS buffer, pH 7.4 at 37°C. The weight change was recorded at different time intervals the equilibrium swelling was reached. The wet weight of the swollen hydrogels was then determined after gently removing the excess liquid using kimwipes. All the experiments were performed by triplicate. The swelling degree was calculated as follows:

(Equation 4) $$\text{Swelling degree }\left(SD\right)=100\times \frac{wt.\;\text{of wet sample}-wt.\;\text{of dried sample}}{wt.\;\text{of dried sample}}$$

#### *In vitro* degradation

Pure and hybrid scaffolds were sterilized and neutralized in alcohol overnight, weighted, washed with 1X PBS buffer at 37°C and then rehydrated with an excess of the same buffer for 24 h before the degradation assay. The enzymatic degradation was carried out at 37°C in 15 mL of PBS (pH 7.4) containing 0.5 mg/mL lysozyme for 28 days. The enzyme solution was refreshed each 3 days to ensure a continuous enzymatic activity. The scaffolds were removed periodically from the medium, washed with distilled water and freeze-dried to determine the dry weight of the remaining polymer. The degradability ratio *D* was calculated as follows:

(Equation 5) $$\text{Degradability }\left(D\%\right)=100\times \frac{wt.\;\text{of sample before degradation test}-wt.\;\text{of sample at specific day}}{wt.\;\text{of sample before degradation test}}$$

Each experiment was conducted in triplicate and the average value was taken as the percentage of degradation.

#### Scaffold sterilization

Prior to use, scaffolds were soaked in a 70% (v/v) ethanol solution for 15 min at room temperature for sterilization. Then, they were washed three times with sterile PBS and incubated in sterile PBS for 15 min at culture conditions for further ethanol clearance and rehydration (37°C, 5% CO_2_). For lixiviate preparations, PBS was discarded and the scaffolds dried, weighted, and incubated in the presence of culture medium for 96 h at culture conditions. For cell culture, PBS was replaced with a complete culture medium and the scaffolds were incubated for a further 30 min at culture conditions for medium impregnation. Then, the polymeric matrices were transferred to culture plates and cells were seeded immediately afterwards.

#### Cell biocompatibility

The biocompatibility of the obtained scaffolds was analyzed in Balb, HaCAT, and MSC cell lines. Two different assays were performed. In the first one, scaffolds' cytocompatibility was determined according to the standard ISO 10993−5:2009 elution method (International Standard, I., 2009) 20 mg of each scaffold were cut into small pieces and immersed in 2 mL of culture medium to produce lixiviates. The lixiviates were incubated for 96 h at culture conditions and added (100 μL of lixiviate solution) to cells that were seeded and incubated for 24 and 72 h in advance (96-well plates, 10000 cells/well, 100 μL of cell suspension). Then, the cell medium was removed and, subsequently, 90 μL of DMEM for Balb and HacaT cells or 90 μL of MesenPRO RS^TM^ for MSC cells and 10 μL of CCK-8 cell proliferation reagent were added, followed by gentle shaking for 1 min and incubation for 1 h. The optical density (OD) of formazan was measured afterwards at 450 nm using an ELISA microplate reader (BIO-RAD model 680, U.S.A.). Cell metabolic activity of cells after exposure to lixiviates, represented as the percentage of cell viability, was calculated by normalizing the formazan OD reading from cells exposed to lixiviates with that from control, non-exposed cells (100% viability) by following the equation:

(Equation 6) $$\text{Cell Viability}\;\%=\frac{Abs\;S\;}{Abs\;B}\;\times 100$$

where Abs S is the absorbance of scaffolds incubated in lixiviate-derived fluid, and Abs B is the absorbance of cells incubated in culture medium. Results were the average of at least three independent experiments.

In the second assay, cell viability was analyzed. Sterilized scaffolds were pre-incubated in DMEM or MesenPRO RS^TM^ for 1 h and placed in 24 well culture plates. Next, 1 mL of cell suspension (50000 cells/well) was injected on top of each scaffold and incubated for 24 and 72 h at 37°C, 5% CO_2_. After incubation, 10 μL of CCK-8 was added to each well and incubated for 1 h. Finally, cell viability was determined by measuring the absorbance at 450 nm using a UV-Vis microplate reader (Bio-Rad model 689, USA) and cell viability was obtained using Equation 6.

#### Cell imaging

Cell adhesion and proliferation were observed by optical microscopy. Cells (50,000 cells in 50 mL of culture medium, 1.0 × 10^6^ cells mL^−1^) were seeded onto scaffolds and maintained for 2 h in an incubator for cell attachment. Next, fully supplemented culture media was added, and cells were cultured for up to 7 days with regular medium changes (ca. each 2 days). Cells were imaged on days 1, 3, and 7 with a Leica DMI6000 fluorescence in both bright field and fluorescence modes. In addition, cells adhered to scaffolds were mounted on top of microscope slides and incubated for 10 min at room temperature and dark conditions in the presence of a calcein AM/propidium iodide solution to be imaged immediately afterwards (98:1:1 of PBS/calcein AM/propidium iodide) for analyzing the viability of adhered cells. Experiments were carried out at least in duplicate for each kind of scaffolds, cell line, and time point. Different zones were imaged. Images were recorded at excitation/emission wavelengths of 480/510 and 560/610 nm for the detection of calcein AM (green) and propidium iodide (red), respectively.

Cell cultured samples for scanning electron microscopy (SEM) analysis were fixed with 2.5% (v/v) glutaraldehyde solution overnight at 4°C. The samples were dehydrated in a series of ethanol washes (0%, 30%, 50%, 70%, 90%, 100% (v/v)), with each one made twice. The samples were critical point dried, sectioned, sputter coated with iridium, and then imaged with a field-emission scanning electron microscopy (FESEM, Zeiss Ultra-Plus, Germany).
